# An Electromyographic Analysis of the Effects of Cognitive Fatigue on Online and Anticipatory Action Control

**DOI:** 10.3389/fnhum.2020.615046

**Published:** 2021-01-11

**Authors:** Mick Salomone, Boris Burle, Ludovic Fabre, Bruno Berberian

**Affiliations:** ^1^Information Processing and Systems, ONERA, Salon de Provence, Base Aérienne 701, France; ^2^Aix-Marseille Université, CNRS, LNC UMR 7291, Marseille, France; ^3^Centre de Recherche de l'Ecole de l'Air, Salon de Provence, Base Aérienne 701, France

**Keywords:** cognitive fatigue, effort, action control, electromyography, gratton effect, inhibition

## Abstract

Cognitive fatigue is a problem for the safety of critical systems (e.g., aircraft) as it can lead to accidents, especially during unexpected events. In order to determine the extent to which it disrupts adaptive capabilities, we evaluated its effect on online and anticipatory control. Despite numerous studies conducted to determine its effects, the exact mechanism(s) affected by fatigue remains to be clarified. In this study, we used distribution and electromyographic analysis to assess whether cognitive fatigue increases the capture of the incorrect automatic response or if it impairs its suppression (online control), and whether the conflict adaptation effect is reduced (anticipatory control). To this end, we evaluated the evolution of the performance over time during the Simon task, a classic conflict task that elicits incorrect automatic responses. To accentuate the presence of fatigue during the Simon task, two groups previously performed a dual-task with two different cognitive load levels to create two different levels of fatigue. The results revealed that time on task impaired online control by disrupting the capacity to suppress the incorrect response but leaving unaffected the expression of the automatic response. Furthermore, participants emphasized speed rather than accuracy with time on task, with in addition more fast guesses, suggesting that they opted for a less effortful response strategy. As the implementation of the suppression mechanism requires cognitive effort, the conjunction of these results suggests that the deficits observed may be due to disengagement of effort over time rather than reflecting an incapacity to make an effort.

## Introduction

Some complex activities, such as piloting an airplane, require a sustained cognitive effort that can lead to cognitive fatigue. This state, which is distinct from drowsiness, can be defined as a difficulty in initiating or sustaining voluntary activities [Adams et al., 1997; for a review see Chaudhuri and Behan (2004)]. There is no consensus on the factors that cause cognitive fatigue. Among the proposed factors, some authors suggest that a decrease in metabolic resources [e.g., glucose; Muraven and Baumeister (2000)] is central while others emphasize the importance of effort and argue that cognitive fatigue should occur when the costs of cognitive effort to perform the activity are higher than the expected benefits (Boksem and Tops, 2008; Kurzban et al., 2013). In this case, after performing an effortful task, disengagement from the current task or unwillingness to sustain the effort on a second task is likely (Inzlicht et al., 2014; Müller and Apps, 2019). However, these two proposals are not mutually exclusive (Christie and Schrater, 2015; André et al., 2019).

Cognitive fatigue can appear in two distinct forms. Changes in performance may be observed (Holtzer et al., 2010). These changes are sometimes referred to as fatigability. Many cognitive processes can be disrupted as cognitive flexibility (Plukaard et al., 2015) or planning (Lorist et al., 2000; van der Linden et al., 2003), which can interfere with the ability to adapt to unexpected situations. Overall, the proper functioning of cognitive control processes appears to be impaired (Lorist and Faber, 2011). Consequently, a decrease in performance can be observed, including an increase in the number of errors (Boksem et al., 2006). Cognitive fatigue can also be subjective, in which case, a feeling of exhaustion or a decrease in motivation can be reported (Gergelyfi et al., 2015). The relation between these two manifestations has often been studied but rarely observed, so they are sometimes considered to be independent (Kluger et al., 2013). Some models suggest that this dissociation is related to the fact that these two manifestations do not appear at the same time. The performance decrement would be later than subjective fatigue because the latter would signal the need to maintain the performance (e.g., Hockey, 2013). But another reason that could contribute to this absence of relation is the lack of sensitivity of the measures used. In this regard, Wang et al. (2014) reported a correlation between trait fatigue perception and the coefficient of variation of RT, but not with other behavioral measures (i.e., RT and accuracy). In addition, only subjective fatigue is generally evaluated but cognitive fatigue is accompanied by other subjective manifestations. Perceived effort is particularly important since cognitive fatigue increases effort costs and when these costs are considered too high, it can lead to disengagement of the task (Hockey, 2013; Inzlicht et al., 2014).

When operators of critical systems (aircraft, nuclear plants, train…) are subject to cognitive fatigue, fatigability can have dramatic consequences. Given the likelihood of the occurrence of cognitive fatigue in the operational context and the role of adaptive capabilities in the safety of these critical systems, it is necessary to understand how cognitive fatigue interferes with the cognitive mechanisms involved in adaptation capabilities and to better understand the relationship between subjective fatigue and fatigability. In this article, we explore this impact through the evaluation of action control during sensorimotor activities.

Action control is defined as the capacity to limit impulsive actions and favor goal-directed ones. Indeed, to adapt to the constraints of a dynamic environment and to limit errors, we must often choose actions adapted to our goals among many others. To this end, two types of control can be distinguished: *online* and *anticipatory* control. These two controls involve different processes and cerebral networks and do not occur at the same time (Ridderinkhof et al., 2011). Online control refers to the processes that inhibit and resist the activation of an automatic and unwanted response for another according to our goals. This control acts after the stimulus presentation and before the incorrect response is emitted. The online control is therefore transitory, changing from trial to trial. Unlike online control, anticipatory control prepares for the correct action. It strengthens the online control or limits its use. Ridderinkhof et al. (2011) consider that anticipatory control can be divided into two parts, *reactive* and *prospective* control [see Braver (2012), for another conception of a dual mechanism of action control]. In the first case, the control is adjusted based on past performance and events (e.g., I strengthen online control after I made a mistake). In the second case, the control is adjusted according to task regularities or instructions, allowing the prioritization of relevant information or anticipating the need for online control.

These two controls, online and anticipatory, have been studied using conflict tasks such as the Simon task (Simon, 1969). In this task, participants must give a lateralized response based on a non-spatial attribute of the stimulus. Although not relevant to the task at hand, the stimulus position automatically activates the hand located ipsilaterally while the relevant attribute activates the hand associated with the instruction. Thus, a conflict may arise when the stimulus is presented on the side opposite to the instruction-based response (incompatible trials). In this case, higher error rates and longer response time (RT) are observed, which is often referred to as the “compatibility effect,” indexing the cost of the automatic activation and its subsequent suppression.

The compatibility effect, however, is sensitive to context. In particular, past events can strongly modulate it. The compatibility effect is largely reduced after an incompatible trial compared to a compatible trial (Gratton et al., 1992; Egner, 2007). This reduction in the compatibility effect after an incompatible trial, called conflict adaptation effect or Gratton effect, is thought to reflect an adjustment of the adaptive control [reactive control; Botvinick et al., 2001, see however Mayr et al. (2003), Hommel et al. (2004) for alternative accounts]. Thus, through the magnitude of the reduction in the compatibility effect and its evolution after an incompatible trial (i.e., the Gratton effect), the Simon task allows the evaluation of both online and anticipatory control mechanisms. Both the mean interference effect and its modulation have been used to assess the origin of cognitive fatigue. We will now briefly review this literature before pointing to the limitation of simply assessing mean behavioral compatibility effects.

Several authors have observed a disruption in online and anticipatory control with cognitive fatigue, but the results are far from being consistent. Concerning online control, in a study requiring the completion of a Simon task for more than 3 h, Möckel et al. (2015) observed an increase in the compatibility effect with time on task (Möckel et al., 2015) suggesting that cognitive fatigue interferes with online control. But the opposite has also been observed in longer studies [Wascher et al., 2014; see also Boksem et al. (2006), Xiao et al. (2015) for similar results]. Studies specifically evaluating the effect of cognitive fatigue on the Gratton effect are relatively scarce, but the same uncertainty seems to apply to anticipatory control in other contexts in which fatigability may be observed. Von Gunten et al. (2018) observed that the Gratton effect remained present throughout an Eriksen flanker task [Von Gunten et al., 2018; see Lorist and Jolij (2012), for similar results]. However, in a sleep-deprived condition in which cognitive fatigue is important, the conflict adaptation effect was impaired, unlike online control (Gevers et al., 2015). Research has mainly focused on another adaptation effect, namely, post-error slowing, but as with online control, inconsistent results have also been observed (Lorist et al., 2005; Boksem et al., 2006; Xiao et al., 2015).

Regarding the different experiments on cognitive fatigue, these inconsistent results may lie in the use of metrics that do not accurately capture the functioning of action control. In this case, cognitive fatigue could be present but its behavioral effects would not be detected by the measures used. Indeed, the use of traditional measures only (e.g., average RT, accuracy) provides only a macroscopic view of the cognitive mechanisms involved in the control of actions.

The size of the compatibility effect (and its modulation) stems from at least two components: the strength of the automatic response activation and the capacity to overcome this initial automatic activation. However, mean compatibility effect measure on behavioral response does not allow to dissociate them. Nevertheless, some tools, however, exist to do so (see below). These tools evidenced that these two mechanisms are largely independent, as they can be specifically affected by different factors [i.e., some factors specifically affect one mechanism, sparing the other one, e.g., see Spieser et al. (2015), Fluchère et al. (2018), Korolczuk et al. (2020) for double dissociations]. Cognitive fatigue could hence either increase automatic activation and/or reduce the capacity to overcome this automatic activation. In this context, measuring these mechanisms separately promises to clarify the impact of cognitive fatigue on the control of the action.

This will be done using electromyographic measures and distribution analysis in a Simon task. The EMG recordings reveal a covert phenomenon evidencing the presence of automatic response activation. On some correct trials, subliminal muscle activation (i.e., that does not exceed the response activation threshold) is observed on the hand muscle associated with the incorrect response before the muscle activation associated with the correct response. Such “partial errors” are more numerous on incompatible trials and reflect (to a large extent) the automatic activation of the incorrect response by the stimulus position (Hasbroucq et al., 1999; Burle et al., 2002). The strength and time course of the automatic response activation can be evaluated by coupling these EMG measures with the conditional incorrect accuracy function, which plots the probability that the first EMG activation is observed in the correct hand, as a function of the latency of this first EMG activation. It is commonly observed that at short latencies, most EMG activations are incorrect on incompatible trials. The percentage of incorrect activations during short trials can be considered as an indicator of the strength of the automatic response activation (Ridderinkhof, 2002; van den Wildenberg et al., 2010). The analysis of the EMG recordings also provides a direct indicator of the suppression mechanism: the ability to overcome incorrect activations can be evaluated by calculating the correction ratio, which is the number of incorrect activations corrected divided by the total number of incorrect activations. A higher correction ratio means a better ability to inhibit incorrect automatic activations (Burle et al., 2002, 2014). Using these two independent measures, we intend to clarify the impact of cognitive fatigue on online control.

In this study, with the association of distribution analysis and EMG, we assessed the extent to which cognitive fatigue impacted online and anticipatory control. Cognitive fatigue has been manipulated in two ways: time on task and using a secondary task, the Time Load Dual Back (TLDB) task (Borragán et al., 2017). The time spent on the task is an important factor leading to cognitive fatigue. To this end, we evaluated the evolution of performance over time during the Simon task. Thus, participants completed a long version (45 min) of the Simon task. Its duration remained shorter than in other studies to limit the involvement of other factors such as boredom or decreased motivation that could explain the performance decline (Möckel et al., 2015). Nevertheless, it remains sufficient since several studies have observed performance decrement with shorter durations (e.g., Lorist et al., 2005). To assess the impact of cognitive fatigue induced by time on task, we will evaluate the measures defined above at the beginning, middle, and end of the experiment. In order to observe whether different levels of cognitive fatigue could be responsible for these differences, we also tried to induce two different levels of fatigue. To this end, our two groups previously performed the TLDB task which quickly induces two levels of cognitive fatigue in two different groups by modulating the cognitive load level of the task (Borragán et al., 2017; O'Keeffe et al., 2020).

Cognitive fatigue primarily affects top-down processes (Lorist and Faber, 2011). If cognitive fatigue disturbs online control, the suppression of the incorrect response (i.e., correction ratio) and/or the strength of the response capture should be impacted over time. For the same reason, the reduction of the compatibility effect after an incompatible trial should be lower over time. These negative effects are expected to be larger for participants who performed the TLDB task with the highest cognitive load, i.e., those for whom we tried to induce even more cognitive fatigue. We also assessed the subjective experience of participants. We made this choice to ensure that cognitive fatigue was induced but also to determine if perceived effort and/or subjective fatigue correlated with EMG measures. Some models indicate that subjective experience precedes the performance decrease (e.g., Hockey, 2013). Thus, we distinguished between perceived effort and subjective fatigue induced by the TLDB task and by the Simon task. Similarly, the accomplishment of a prolonged task can modulate other subjective manifestations like sleepiness and alertness. In addition, we will also control the evolution of these variables. Our hypotheses are therefore that subjective fatigue increases over time and that this increase, along with perceived effort, is greater for participants who performed the TLDB task with the highest cognitive load. Since the total duration of the study is important, we also expect an increase in sleepiness and a decrease in alertness with time on task. However, we should observe a correlation only between EMG measures and subjective fatigue and perceived effort, but not with sleepiness and alertness.

## Method

### Participants

Twenty four participants volunteered for this study and were randomly assigned to one of two groups differing in the amount of cognitive fatigue induced (see below). The “High Cognitive Load” group (HCL) was composed of 12 participants (3 men, M = 22; SD = 2.74) and the “Low Cognitive Load” group (LCL) as well. In this group, however, one participant's data could not be used due to a technical problem (3 men, M = 22.1; SD = 2.55). All participants had normal or corrected-to-normal vision and reported no history of psychiatric or neurological disease. They were paid 10 Euros/h. This experiment was approved by the Comité de Protection des Personnes Sud Méditerranée 1 (approval 1041). Participants gave their informed written consent according to the Declaration of Helsinki.

### Materials

Participants were seated in a comfortable chair 70 cm in front of a CRT monitor with a refresh rate of 70 Hz and a screen resolution of 1,024 × 768. They were tested in a dark, sound-shielded Faraday cage. PsychoPy software (Peirce, 2007) was used to display stimuli and to collect behavioral and subjective data. Responses were made by pressing either a left or a right button with the corresponding thumb. The buttons were fixed to the tops of two plastic cylinders (3 cm in diameter, 9 cm in height) separated by 20 cm. Button releases were transmitted to the parallel port of the recording PC to reach high temporal precision.

### Tasks Performed by the Participants

Participants performed different tasks, which will first be described separately. The time course of the different tasks will then be presented.

#### The Time Load Dual Back (TLDB) Task

This task is a dual-task combining a parity judgment task and an N-back task [see Borragán et al. (2017) for more details on the task]. Letters (A, C, T, L, N, E, U, and P) and digits (1 to 8) were displayed (Arial, size = 2°) in alternation. Participants were asked to indicate whether the digit was odd or even by pressing either the right or the left button and whether the displayed letter was the same as the penultimate letter (2-back task) by pressing either the right or the left button again. The response mapping was counterbalanced across participants. The task was divided into several blocks of 30 letters and 30 digits pseudo-randomly presented. In each block, there were 10 target letters. The number of blocks depended on the stimulus duration (STD) which was set individually for each participant to adjust the cognitive load. The computation of the individual STD was performed during a pre-test session on a different day from the test session. During this pre-test session composed of four tasks, participants were first trained on each task separately, then on the combination of the two (i.e., the core TLDB task) and finally the individual STD was computed during another TLDB task. The STD was initially set to 2,000 ms for the three training tasks. Training tasks stopped if the accuracy of the participants was more than 85%^1^. over a block. To compute the STD for each participant in the fourth task, the STD was set to 1,900 ms in the first block and if the accuracy score was ≧85%, the STD decreased by 100 ms for the next block. To reduce the duration of the pre-test session, the STD decreased by 200 ms if the accuracy score was ≧95%. This task was again interrupted when the accuracy dropped below 85%. The STD of the last successful block was assigned to the HCL condition. The STD in the LCL condition was made 50% longer than in the HCL condition. Regardless of the STD, the duration of the task lasted ~24 min^2^. There was a slight variation to allow for the completion of the ongoing block. In all tasks, participants were instructed to respond quickly and accurately.

#### The Simon Task

Participants completed a training session of 48 trials and a test session of 15 blocks of 96 trials each. The blocks were separated by a break of up to 1 min. Each trial started with the apparition of a white fixation cross for 500 ms. Then a circle (diameter = 1.4°) red (RGB: 0.835, −1, −1) or blue (−1, −1, 0.835) was displayed at 3° to the left or right of the fixation cross and disappeared after 1,000 ms if no response was given. Half of the participants were asked to answer with their right hand when the circle was blue and with their left hand when the circle was red. The response mapping was reversed for the other half of the participants. An inter-trial interval of 500 ms ended the trial. Half of the trials were compatible which means that the stimulus was displayed on the same side as the required response, and the other half were incompatible (stimulus displayed on the opposite side to the required response). The trials were pseudo-randomized using Mix software (van Casteren and Davis, 2006) so that the compatibility sequences (i.e., compatible–incompatible CI, CC, IC, and II) occurred the same number of times. Participants were asked to respond as quickly and accurately as possible according to the color of the circle and regardless of its position.

#### Psychomotor Vigilance Task (PVT)

The purpose of this task was to assess each participant's vigilance level before the test. We focused on two measures that appear to be sensitive enough to detect low vigilance level (Basner and Dinges, 2011). We counted the number of omissions (i.e., RT > 500 ms) and the inverse of the RT. The duration of the task was 5 min. Each trial started with a timer being triggered after a random delay of between 2 and 10 s. The participant's task was to click on a mouse button as quickly as possible to stop the timer.

### Subjective Scales

Usually, when a task is performed over a long time, different subjective manifestations can appear. Therefore, several scales measuring different constructs were used. Subjective fatigue and sleepiness were measured by two visual analog scales [VASf and VASs, respectively; Lee et al. (1991)]. We also used the Samn-Perelli scale, which instead measures the level of alertness (Samn and Perelli, 1982). We also measured the cognitive load level that participants assigned to different tasks with the NASA RTLX (Hart, 2006). This scale is composed of six subscales assessing mental demand, physical demand, temporal demand, performance, effort, and frustration. In this study, apart from the evaluation of the average of the subscales, we focused on the subscale “effort” to evaluate the correlation between objective measures and perceived effort. Other measures such as mental demand could have been included but it mainly reflects the difficulty of the task.

### Procedure

**Figure 1:**
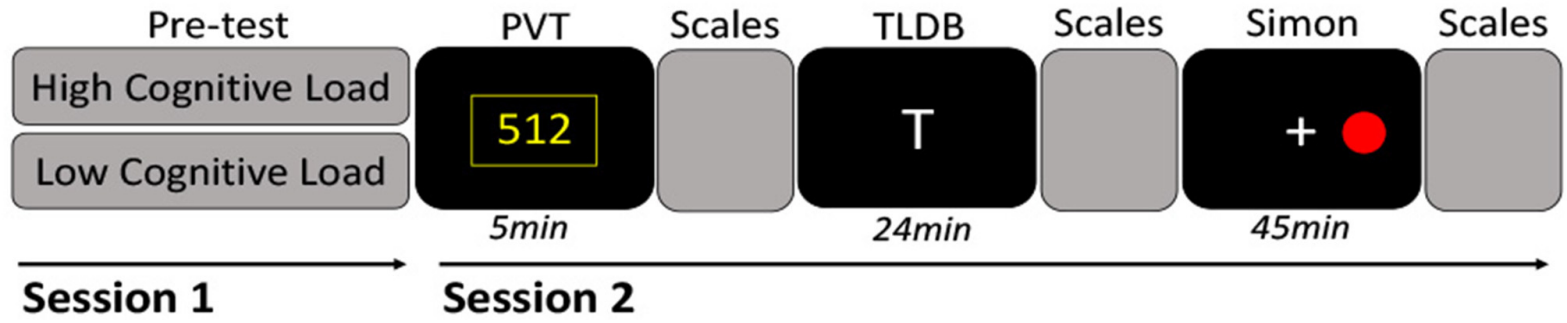
Procedure. On the first session, the participants completed a pre-test to adapt the cognitive load level of the TLDB task according to their capacity. During the second session, which was scheduled on a different day, participants first performed the PVT to assess their vigilance level. Before (time 1) and after the TLDB task (time 2) and the Simon task (time 3), several scales were filled: the Visual Analogic Scale of cognitive fatigue (VASf) and sleepiness (VASs), the Samn-Perelli, and the NASA RTLX (only after each of these two tasks). The TLDB task was performed either in a high (HCL) or low (LCL) cognitive load. The Simon task was the same for both conditions. This second session lasted ~80 min.

The study was divided into two sessions. The first session was the pre-test session. Participants were trained to perform the TLDB task and the STD was evaluated for each participant. In the second session, they performed the tasks in the following order: the PVT, the TLDB task (either in the high or low cognitive load condition), and the Simon task. The scales (i.e., VASf, VASs, and the Samn-Perelli) were completed before and after the TLDB task and the Simon task (i.e., three times in the experiment) while the NASA RTLX was filled out only after these two tasks (Figure 1). The average delay between the two sessions was 2.6 days (*SD* = 1.9). As far as possible, the two sessions were completed at the same time of day on different days. The sessions took place between 8:00 a.m. and 12:00 a.m. and between 2:00 p.m. and 6:00 p.m. Each participant was asked to have enough sleep the night before the experiment. They were not aware of the duration of the tasks and could not make an objective evaluation during the test.

### EMG Recordings and Processing

The EMG activity of the flexor pollicis brevis from both hands was recorded with two surface Ag-AgCl electrodes (Biosemi, Amsterdam, The Netherlands) fixed ~2 cm apart on the thenar eminences. The sampling rate was 2,048 Hz and the signal was high-pass filtered off-line at 10 Hz. The EMG signal was continuously monitored by the experimenter to avoid, as far as possible, any background activity that might interfere with the signal recording and mask small muscle activations. In the case where tonic muscular activity was observed or during the breaks between blocks, the experimenter asked the participant to relax their muscles. The EMG onsets were hand-scored after visual inspection. This method took longer than the automated algorithm, but the recognition of small muscle activations is better (Staude et al., 2001).

### Data Analysis

**Figure 2:**
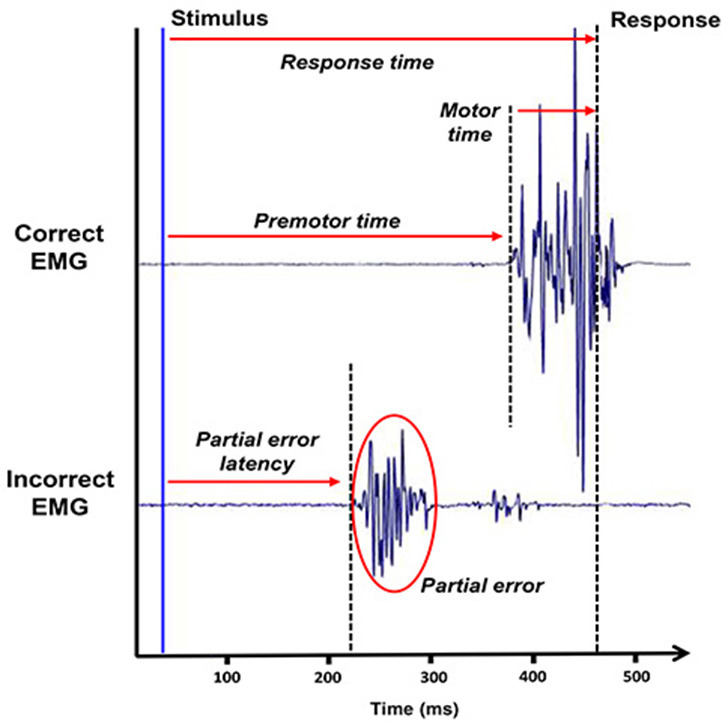
The chronometric measures recorded during this task. The electromyographic recording of the two agonists associated with the two possible responses as a function of time (in ms) allows to observe partial errors and to distinguish different chronometric measures. It enables the separation of the classical response time into two different measures that we used in this study, the pre-motor time and the motor time.

Anticipations (trials with RT <100 ms) were excluded from the analysis for both tasks. Trials of the Simon task were classified into three categories. The correct trials were separated according to whether an EMG burst was recorded (partial-error trials) or not (pure-correct trials) on the incorrect side preceding the correct response. Trials were defined as errors when only the incorrect response was recorded. Trials that did not correspond to these three categories were rejected from the analysis. A total of 12.8% of the trials were excluded. From the distinction between these three categories, we were able to extract several variables (Figure 2). First, the RT was fractionated into different intervals: for all trials, we defined the pre-motor time (from stimulus presentation to correct EMG onset) and motor time (from EMG onset to mechanical response recording). For trials containing a partial error, a third chronometric variable was extracted: the partial error latency, which corresponds to the time from stimulus presentation to the onset of the incorrect EMG burst. Second, errors and partial errors were also extracted to compute the conditional incorrect accuracy function and the correction ratio. The conditional incorrect accuracy function was constructed by taking the first EMG activation, whether correct or incorrect, and spitted the distribution into five bins with the same number of trials. For each bin, we computed the proportion of correct EMG and the mean value of the latencies of this bin. The proportion is then plotted as a function of the mean bin latency to construct the conditional incorrect accuracy function. To evaluate anticipatory control, we analyzed the Gratton effect. Trials were classified according to the compatibility of the preceding trial. For this analysis, the first trial in each block was excluded and all *n* trials were correct trials. The *N*-1 trials were correct trials when we analyzed RT.

### Statistical Analysis

We proceeded in several steps for the statistical analysis. First, we analyzed the control variables to confirm that the two groups were equal in various aspects at the beginning of the test session, such as their level of alertness and their performance during the pre-test session. These different measures were subjected to an analysis of variance (ANOVA) with cognitive load level (HCL or LCL) as a between-subject factor.

We wanted to quantify the evolution over time of the different subjective experiences (e.g., subjective fatigue, sleepiness) felt by the participants during the experiment. Thus, we analyzed separately the evolution of scores after the TLDB task and after the Simon task. But first, we looked at the subjective ratings of the participants at the beginning of the test. It is important to assess whether participants were already tired or sleepy, as this could have a significant impact on performance and ratings. The data were submitted to multiple ANOVAs. The score from each of the different scales (i.e., VASf, VASs, Samn-Perelli, and NASA RTLX) was used as a dependant variable. The between-subject factor “cognitive load level” (HCL or LCL) was again included and, when necessary, the within-subject factor “time,” referring to the different times the questionnaire was completed.

As explained above, one of the objectives of the TLDB task was to increase the presence of cognitive fatigue during the Simon task, which was already expected to be caused by the time spent on the task. In addition to assessing the evolution of subjective fatigue after performing the TLDB task, we evaluated the evolution of performance during this task. Observing a different decline in performance over time between the two groups would ensure that the task fulfilled its role. To this end, the TLDB task was divided into six blocks and the two sub-tasks were evaluated separately during the analyses. We extracted two behavioral markers (RT and accuracy) to explore whether changes in performance were observed across blocks (within-subject factor, block 1 to 6) and/or as a function of cognitive load level (between-subject factor, HCL or LCL).

Finally, to characterize online and anticipatory control, we combined the classical measures of the compatibility effect (i.e., RT, accuracy, and the Gratton effect) with complementary measures (pre-motor time, motor time, incorrect activation rate, conditional incorrect accuracy function, correction ratio) only accessible with EMG recordings. By using such measures, we wanted to clarify how cognitive fatigue affected automatic response activation, suppression mechanisms, and anticipatory control when performing the Simon Task. To assess the evolution of these measures as a function of time on task, blocks were included as a within-subject factor. We grouped the 15 blocks into 3 large blocks and compared only blocks 1 and 3 (i.e., the first 5 and last 5 blocks). The trial sequences were considered as a within-subject factor in the evaluation of the Gratton effect. For the conditional incorrect accuracy function analysis, the bin variable was added as a within-subject factor. An appropriate transformation was applied to the chronometric variables to meet the conditions of application of the ANOVA. The percentages were specifically submitted to arcsine transformation because it stabilizes the variance (Winer, 1962). Multiple pairwise comparisons were carried out with *p*-values adjustment using Tukey's method. In addition to *p*-values, the partial eta square was reported to assess relationships within the data.

To finish, Pearson correlations between objective and subjective measures were computed and *p*-values were corrected for multiple comparisons using the Bonferroni correction.

## Results

### Control Variables and Pre-test

Analyses conducted on the two indicators of the PVT (i.e., number of omissions and the inverse of the RT) did not reveal any differences between the two groups, *Fs* < 1. We also analyzed whether the delay between pre-test and test session was equal between the two groups. It averaged 2.6 days (*SD* = 1.9) and there was no difference between the groups (*p* > 0.1). Finally, we controlled whether there was a difference between the two groups on the STD to ensure that neither group was better on the TLDB task. The STD (mean = 1,669; SD = 294) were statistically equivalent according to the cognitive load level (*p* > 0.1).

### Subjective Scales

#### Beginning of the Test

To ensure that both groups reported equal levels of subjective fatigue, alertness, and sleepiness at the start of the task, we compared subjective assessments at the beginning of the study. On all scales, there was no difference between the two groups, *Fs* < 2.94, *ps* > 0.1.

#### The TLDB Task

To assess the evolution of the different subjective experiences induced by this task, we compared the scores of the scales completed before and after its completion. Regarding the two visual analog scales and the Samn–Perelli scale, the analysis showed an increase of their scores over time, *Fs*_(1, 21)_ > 7.8, *ps* < 0.01, $\eta_{p}^{2}$ > 0.27. However, no interaction was observed, *Fs*_(1, 21)_ < 2.9, *ps* > 0.1, $\eta_{p}^{2}$ < 0.12. This result suggests that it partially had the desired effect as we observed an increase of subjective fatigue equally for both groups. Indeed, we expected higher subjective fatigue in the HCL group. We also observed a main effect of cognitive load on the VASs score, *F*_(1, 21)_ = 4.5, *p* < 0.05, $\eta_{p}^{2}$ = 0.18, with HCL participants reporting higher levels of sleepiness. In other words, while the TLDB generated an increase in the subjective fatigue level, it seems that the manipulation of the cognitive load did not induce two different levels of fatigue. Finally, the mean scores obtained on the NASA RTLX scale were equal for both groups, *F*_(1, 21)_ = 1.05, *p* > 0.1, $\eta_{p}^{2}$ = 0.05. This result indicated that the participants attributed the same level of cognitive load to both tasks.

#### The Simon Task

This time we compared the scores before and after the Simon task. As after the TLDB task, participants in both groups reported a similar increase over time in scores on both visual analog scales and the Samn-Perelli scale, *Fs*_(1, 21)_ > 8.2, *p* < 0.01, $\eta_{p}^{2}$ > 0.28. No main effect of cognitive load or interaction between the two factors was observed, *Fs*_(1, 21)_ < 2.9, *ps* > 0.1, $\eta_{p}^{2}$ > 0.12. These results suggest that the level of subjective fatigue, sleepiness, and alertness continued to evolve in the same direction during the Simon task, regardless of the cognitive load initially used. Finally, participants in both groups reported the same level of cognitive load, *F*_(121)_ = 2.4, *p* > 0.1, $\eta_{p}^{2}$ = 0.1.

#### The Whole Study

We compared the scores at the beginning and the end of the study. Participants reported an increase over time in scores on both visual analog scales and the Samn-Perelli scale, *Fs*_(121)_ > 18.7, *ps* < 0.001, $\eta_{p}^{2}$ > 0.47. No effect of cognitive load, *Fs*_(1, 21)_ < 2.2, *ps* > 0.1, $\eta_{p}^{2}$ < 0.09, or interaction between cognitive load or time was observed, *Fs* < 1.

To sum up, these analyses indicated that subjective fatigue increased after the TLDB task and again after the Simon task. As expected, we observed that the TLDB task was effective in inducing subjective fatigue and we observed the presence of a time on task effect during the Simon task. However, the additional cognitive load in the HCL condition appears to have no impact on the level of subjective fatigue. The scores of each scale are presented in Table 1.

**Table 1:** Subjective ratings for each scale according to the group, and the completion time.

	**Scale**				
	**High/Low cognitive load**				
**Completion time**	**VASf**	**VASs**	**Samn-Perelli**	**NASA RTLX**	**NASA RTLX–Effort**
Time 1	30.7/20.6	33.2/18.5	3.31/3		
Time 2	58.5/40.3	50.4/34.6	4.3/3.3	55/50	67.6/66.6
Time 3	67.4/55.9	59.2/56	4.9/4.4	53.8/40.9	63.9/59.5

*VASf/s, visual analog scale evaluating cognitive fatigue/sleepiness*.

### The TLDB Task

Concerning RTs, on average participants were equally fast to respond during the two sub-tasks, *Fs* < 1. A main effect of block was observed on the 2-back task, *F*_(5,105)_ = 2.9, *p* = 0.05, $\eta_{p}^{2}$ = 0.12, and on the parity judgment task, *F*_(5,105)_ = 6, *p* < 0.0001, $\eta_{p}^{2}$ = 0.22. *Post-hoc* pairwise comparisons between the different blocks revealed a decrease in RT between the second and the last block (block2: 757 ms, block 6: 704 ms) during the 2-back task, *t*_(110)_ = 3.04, *p* < 0.05, and between the first and last block during the judgment parity task (block 1: 701 ms, block 6: 651 ms), *t*_(110)_ = 4.3, *p* < 0.001. The interaction of the factors was not significant for both tasks, *Fs* < 1.

Regarding accuracy, during the 2-back task participants in the LCL condition were more accurate than participants in the HCL condition (91 vs. 85%), *F*_(1, 21)_ = 8.5, *p* < 0.01, $\eta_{p}^{2}$ = 0.29. They were also better during the judgment parity task (98 vs. 95%), *F*_(1, 21)_ = 9.7, *p* < 0.01, $\eta_{p}^{2}$ = 0.32. This first analysis confirmed that the TLDB task performed by the HCL group was more difficult. In addition, the number of errors committed by both groups was stable across blocks in the 2-back task, *F*_(5, 105)_ = 1.5, *p* > 0.1, $\eta_{p}^{2}$ = 0.07, and in the parity judgment task, *F* < 1. These two-way interactions between cognitive load and blocks were not significant for both tasks, *F*_(5,105)_ = 1.4, *p* > 0.1, $\eta_{p}^{2}$ = 0.06, (2-back task), *F*_(5,105)_ = 1.3, *p* > 0.01, $\eta_{p}^{2}$ = 0.06 (judgment parity task). We cannot infer from this result that cognitive fatigue was induced due to the stability of the performance relative to the time on task.

To summarize, while the analysis of the subjective measures seems to indicate that the TLDB task increases subjective fatigue over time, analysis of behavioral indicators show no degradation of performance over time. On the contrary, the decrease in RT over time suggests a learning effect. Crucially, the TLDB task failed to induce two different levels of cognitive fatigue both at the subjective and behavioral levels.

### Effects of Cognitive Fatigue on Online Control During the Simon Task

Descriptive statistics of the behavioral measures assessed in the Simon task are presented in Table 2.

**Table 2:** Descriptive statistics of the behavioral measures assessed in the Simon task.

	**Block 1**				**Block 2**				**Block 3**			
	**Compatible**		**Incompatible**		**Compatible**		**Incompatible**		**Compatible**		**Incompatible**	
	**HCL**	**LCL**	**HCL**	**LCL**	**HCL**	**LCL**	**HCL**	**LCL**	**HCL**	**LCL**	**HCL**	**LCL**
Acc (%)	95 (1)	96 (1)	93 (2)	94 (1)	95 (1)	97 (1)	91 (2)	93 (1)	95 (2)	97 (1)	90 (1)	92 (1)
RT (ms)	356 (19)	344 (13)	373 (20)	365 (14)	355 (20)	344 (14)	372 (20)	368 (15)	353 (19)	341 (12)	375 (22)	364 (13)
PMT (ms)	222 (12)	228 (11)	241 (13)	249 (11)	216 (14)	222 (13)	236 (14)	245 (14)	212 (12)	217 (10)	235 (15)	240 (11)
MT (ms)	134 (9)	161 (8)	132 (9)	116 (8)	139 (9)	122 (7)	136 (9)	123 (7)	141 (9)	124 (6)	140 (9)	123 (6)
IA (%)	19 (2)	15 (1)	37 (3)	32 (2)	20 (2)	16 (2)	39 (3)	34 (1)	21 (1)	18 (2)	40 (2)	37 (3)
CR (%)	76 (4)	78 (5)	82 (5)	80 (3)	78 (5)	78 (4)	78 (4)	79 (4)	78 (4)	83 (4)	74 (5)	79 (3)
	**CC**	**CI**	**IC**	**II**	**CC**	**CI**	**IC**	**II**	**CC**	**CI**	**IC**	**II**
RT (HCL)	346 (18)	387 (22)	366 (20)	360 (19)	345 (19)	378 (22)	365 (22)	363 (19)	347 (18)	382 (23)	357 (20)	367 (20)
RT (LCL)	335 (13)	375 (14)	352 (12)	357 (14)	334 (13)	374 (17)	354 (15)	362 (13)	332 (12)	369 (14)	348 (12)	354 (12)
Acc (HCL)	86 (2)	45 (4)	72 (3)	68 (4)	82 (2)	43 (4)	70 (3)	65 (3)	81 (2)	43 (4)	68 (1)	62 (4)
Acc (LCL)	89 (2)	53 (2)	75 (2)	77 (3)	87 (3)	51 (3)	77 (2)	72 (2)	84 (2)	46 (3)	74 (3)	67 (4)

*Mean (standard error). HCL, High Cognitive Load; LCL, Low Cognitive Load; RT, Response Time; Acc, Accuracy; PMT, Pre-motor time; MT, Motor time; IA, Incorrect Activation rate; CR, Correction ratio; CC, Compatible–Compatible; CI, Compatible- Incompatible; IC, Incompatible–Compatible; II, Incompatible–Incompatible*.

#### Classical Measures (RT and Accuracy)

Participants in the HCL group were not faster than participants in the LCL group, *F* < 1. RT was not modulated through blocks, *F* < 1. The compatibility effect was present (compatible: 349 ms; incompatible: 369 ms), *F*_(1, 21)_ = 140.3, *p* < 0.0001, $\eta_{p}^{2}$ = 0.87, but was not different between the two groups, *F* < 1, nor between blocks, *F*_(1, 21)_ = 1.2, *p* > 0.1, $\eta_{p}^{2}$ = 0.05. The interaction of all these factors was not significant, *F* < 1.

As for the RT, accuracy rate was statistically equal for both groups, *F* < 1, and did not decrease across blocks, *F*_(1, 21)_ = 1.3, *p* > 0.1, $\eta_{p}^{2}$ = 0.061. The compatibility effect was again observed (compatible: 96%; incompatible: 92%), *F*_(1, 21)_ = 37.9, *p* < 0.0001, $\eta_{p}^{2}$ = 0.64. It was also identical for both groups, *F* < 1, but as for RT, it increased through blocks (block 1: 3%; block 3: 5%), *F*_(1, 21)_ = 24.8, *p* < 0.0001, $\eta_{p}^{2}$ = 0.54. This evolution was not modulated by cognitive load, *F* < 1.

In summary, these results indicate that (1) the compatibility effect was present in both conditions, (2) it increased with time on task but (3) it was no different relative to the cognitive load level of the previous task. At this stage, we were unable to differentiate the role of automatic response activation and suppression mechanisms in the observed effect. The use of EMG measurements was intended to address this limitation.

#### Pre-motor Time and Motor Time

In order to determine whether cognitive fatigue influences decision and/or execution time, the latencies of pre-motor time and motor time were separated in the analyzes.

The pre-motor time was higher in incompatible trials compared to compatible trials (241 vs. 220 ms), *F*_(1, 21)_ = 234.2, *p* < 0.0001, $\eta_{p}^{2}$ = 0.92. It decreased with time on task (235 vs. 226 ms), *F*_(1, 21)_ < 13.7, *p* < 0.001, $\eta_{p}^{2}$ = 0.39 but was not modulated by cognitive load (228 vs. 234 ms for HCL and LCL group, respectively), *F* < 1. Moreover, no interaction was significant, *Fs* < 1.

**Figure 3:**
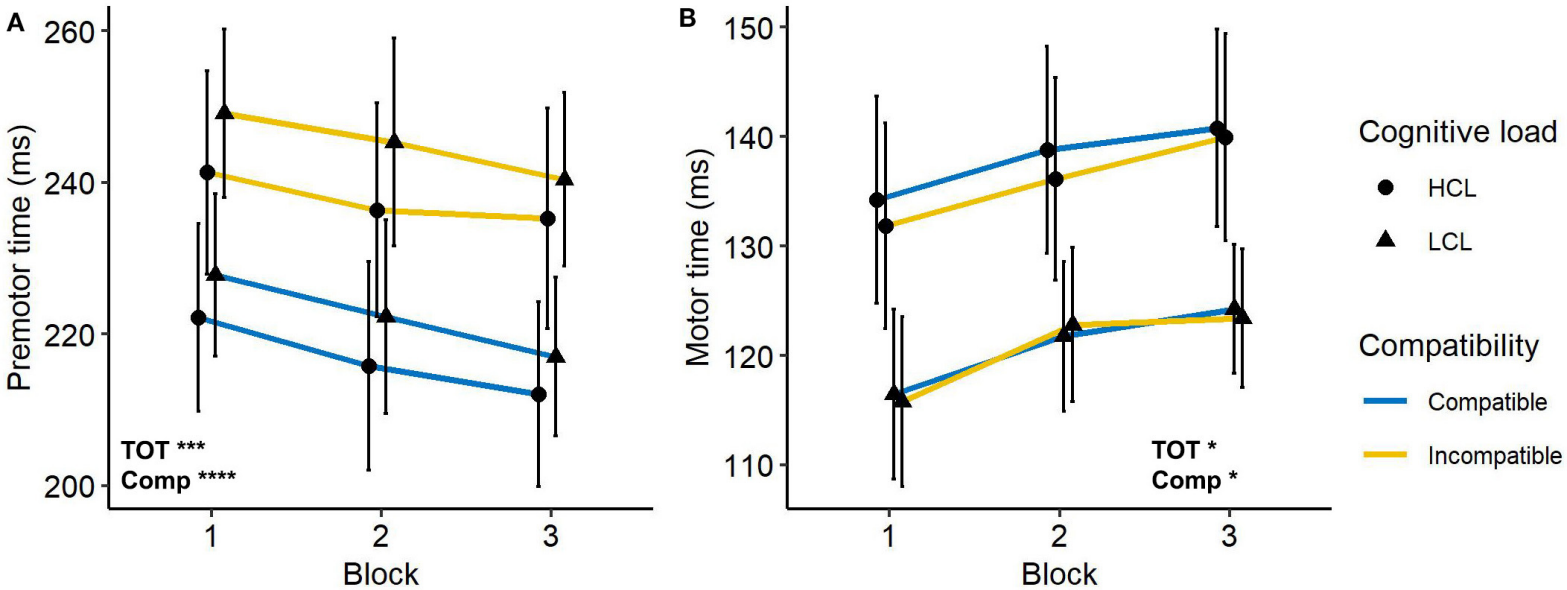
**(A)** Pre-motor time and **(B)** motor time as a function of trial compatibility, cognitive load, and block. Error bars represent the standard error. TOT, main effect of Time-on-Task; Comp, main effect of Compatibility. *p < 0.05; ***p < 0.001; ****p < 0.0001.

We also observed a compatibility effect on motor time. It was higher for compatible trials (129 vs. 128 ms), *F*_(1, 21)_ = 5.6, *p* < 0.05, $\eta_{p}^{2}$ = 0.21. It increased with time on task (125 vs. 132 ms), *F*_(1, 21)_ = 4.5, *p* < 0.05, $\eta_{p}^{2}$ = 0.18, but with the same extent for the two groups, *F*_(1, 21)_ = 2.2, *p* > 0.1, $\eta_{p}^{2}$ = 0.16. All interactions were not significant, *Fs* < 1. These measures are illustrated in Figure 3.

The decomposition of the RT into pre-motor time and motor time reveals that these two indicators were affected by cognitive fatigue and had an opposite dynamic with time on task.

#### Incorrect Activation Rate and Correction Ratio

An increase in the number of incorrect automatic activations only during incompatible trials with cognitive fatigue would correspond to an increase in response capture while a decrease in the correction ratio would inform on the disruption of the suppression mechanism.

Participants made more incorrect activations in incompatible trials (35 vs. 18%), *F*_(1, 21)_ = 261.5, *p* < 0.0001, $\eta_{p}^{2}$ = 0.93. In addition, compared to the first block, more incorrect activations were found during the last block (25 vs. 28%), *F*_(1, 21)_ = 11.8, *p* < 0.01, $\eta_{p}^{2}$ = 0.36. But there was not a main effect of cognitive load, *F*_(1, 21)_ = 2.3, *p* > 0.1, $\eta_{p}^{2}$ = 0.1. The difference observed according to the trial compatibility of the trial was not influenced by cognitive load, *F* < 1, or by blocks, *F* < 1. The interaction of the three factors was also not significant, *F* < 1. This analysis highlighted that, contrary to time on task, the cognitive load level of the TLDB task did not change the number of incorrect activations. However, they increased in both types of trials, whereas we expected an increase only during incompatible trials. The presence of incorrect activations during compatible trials can be interpreted as fast guesses. Thus, this result cannot be fully interpreted as an increase in the capture of incorrect responses over time because of the presence of fast guesses during compatible trials.

**Figure 4:**
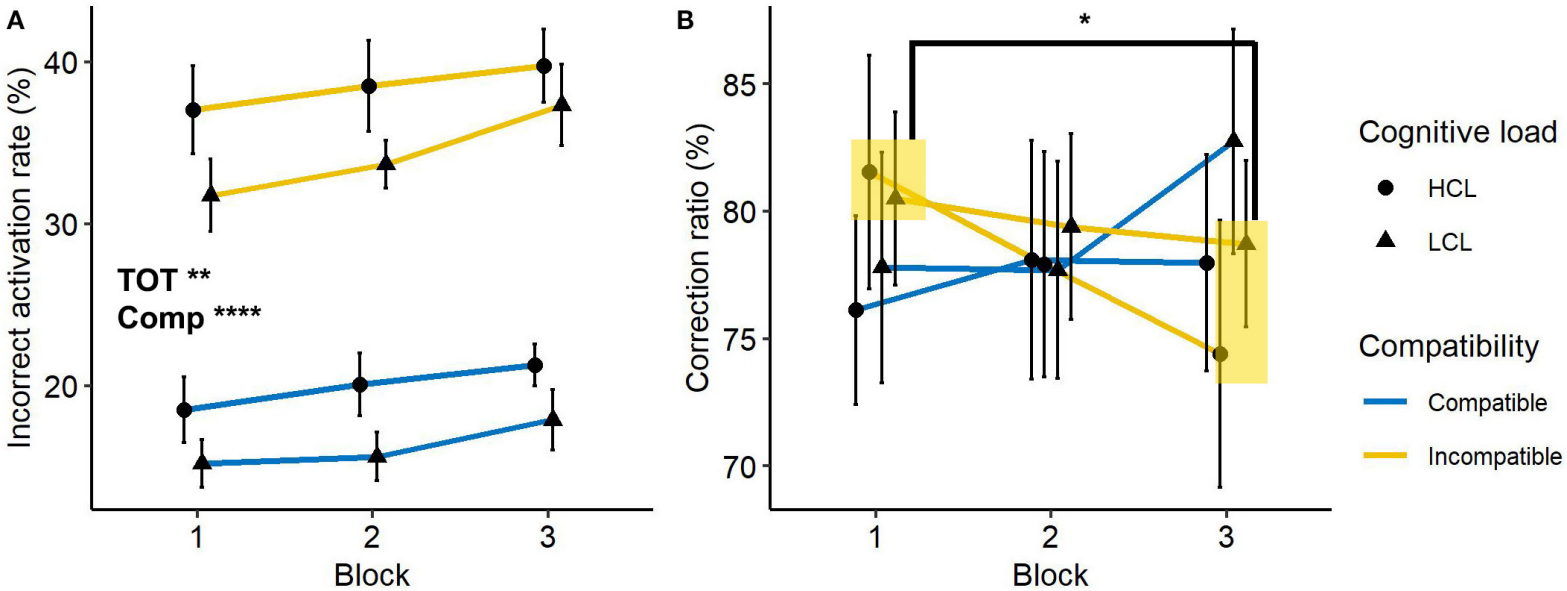
**(A)** Incorrect activation rate and **(B)** correction ratio as a function of trial compatibility, cognitive load and block. Error bars represent the standard error. TOT, main effect of Time-on-Task; Comp, main effect of Compatibility. *p < 0.05; **p < 0.01; ****p < 0.0001.

Analysis on the correction ratio showed that no effect was significant, *Fs* < 1, except the interaction indicating a change in the compatibility effect through blocks, *F*_(1, 21)_ = 19.6, *p* < 0.001, $\eta_{p}^{2}$ = 0.48. The correction ratio on compatible trials remained stable across blocks (77 vs. 80%), *F*_(1,22)_ = 3.5, *p* > 0.1, $\eta_{p}^{2}$ = 0.14, while it decreased on incompatible trials (81 vs. 76%), *F*_(1,22)_ = 6.4, *p* < 0.05, $\eta_{p}^{2}$ = 0.22. Besides making more errors, participants were also less able to correct them during an incompatible trial with time on task. These measures are illustrated in Figure 4.

Taken together, our results indicate that time on task affects both incorrect activation and correction ratio, whereas no effect of cognitive load was observed. Critically, incorrect activations appear to be impacted in both compatible and incompatible trials.

#### Distributional Analysis—The Conditional Incorrect Accuracy Function

**Figure 5:**
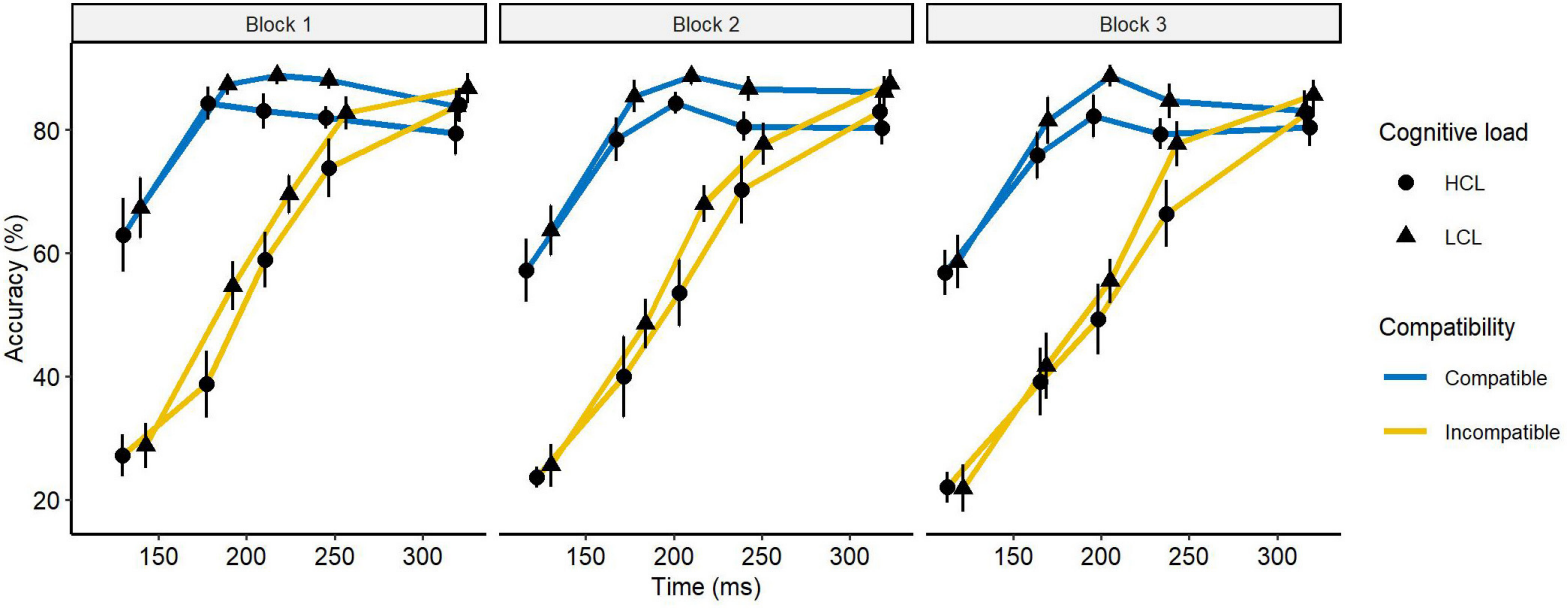
Conditional incorrect activations function for compatible and incompatible trials as a function of cognitive load and block. Error bars represent the standard error.

The conditional incorrect accuracy function aimed to explore the strength and the time course of the automatic response activation. An increase in this strength with time on task should be observed in the first bins. Examination of the conditional incorrect accuracy function (Figure 5) revealed a significant interaction between compatibility and bins, indicating an uneven distribution of the compatibility effect between the different bins, *F*_(4,84)_ = 57.7, *p* < 0.0001, $\eta_{p}^{2}$ = 0.73. Multiple pairwise comparisons revealed that the compatibility effect was higher in the first bin than in the fourth (40 vs. −4%), *t*_(88)_ = 7.9, *p* < 0.001). This effect was most pronounced in the second bin (44 vs. −4%). Statistically, it was no larger than the effect observed in the first bin, *t*_(88)_ = −1.05, *p* > 0.1), but larger than the effect observed in the third bin, *t*_(88)_ = 2.97, *p* < 0.05). These results confirmed that a response capture occurred because the compatibility effect was higher during short trials and equalized as the pre-motor time lengthened. This interaction was not modulated by cognitive load, *F* < 1, but by blocks, *F*_(4,84)_ = 2.8, *p* < 0.05, $\eta_{p}^{2}$ = 0.12. We isolated the first and the second bins to see if the interaction was still present, but it was not, *Fs* < 1. The interaction of all these factors was not significant, *F*_(4, 84)_ = 1.9, *p* > 0.1, $\eta_{p}^{2}$ = 0.08. The result of this analysis suggests that the strength of the automatic response remains the same without being modified by cognitive load or time on task.

In summary, our assessment of online control suggests that the increase over time in the number of incorrect activations during incompatible trials is caused by a suppression deficit since the strength of the automatic response remains stable over time. On the other hand, response capture does not appear to be impacted by time on task.

### Effects of Cognitive Fatigue on Anticipatory Control

**Figure 6:**
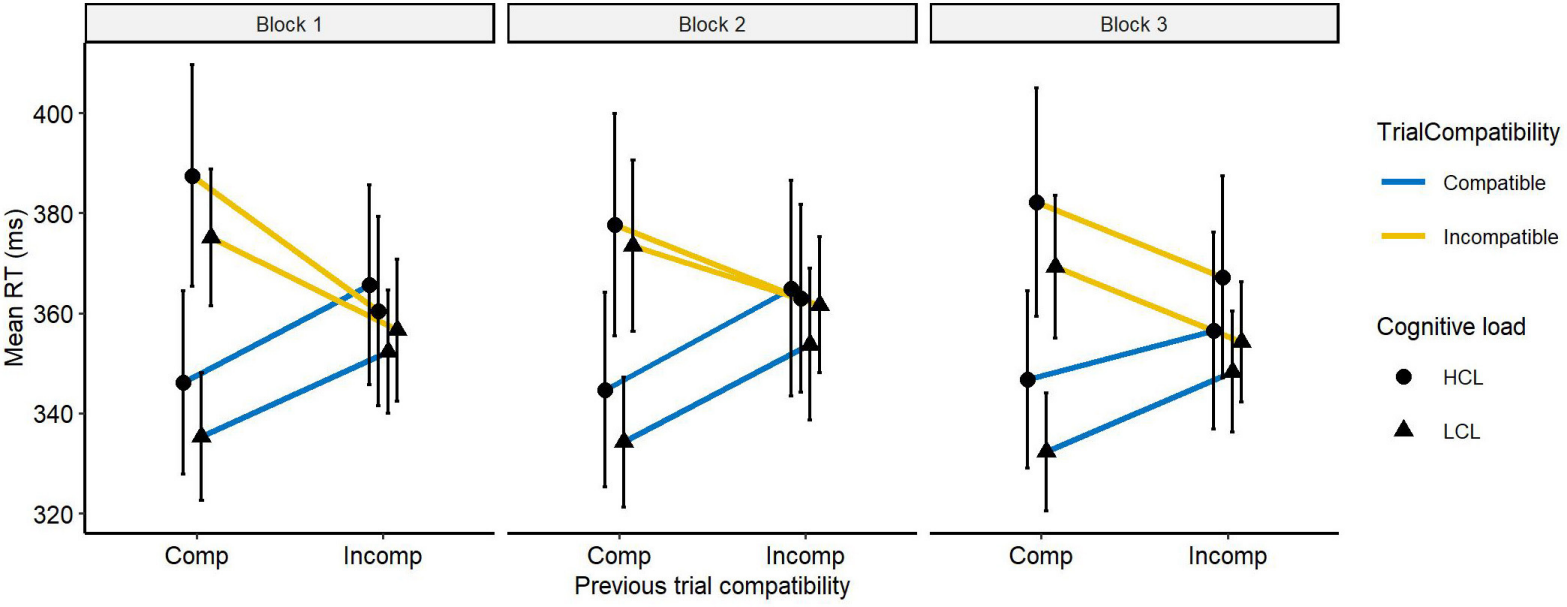
Response time (in ms) as a function of cognitive load, trial compatibility (compatible and incompatible), and compatibility of the previous trial (compatible and incompatible). Error bars represent the standard error.

To evaluate the effect of cognitive fatigue on anticipatory control, we analyzed the evolution of the Gratton effect. A disruption in anticipatory control should be evidenced by an increase in the compatibility effect after an incompatible trial with time on task (Figure 6). A first analysis on accuracy revealed a larger compatibility effect after a compatible trial than after an incompatible trial (39 vs. 4%), *F*_(1, 21)_ = 124.9, *p* < 0.0001, $\eta_{p}^{2}$ = 0.86. Thus, according to the literature, participants adapted their behavior after an incompatible trial resulting almost by the disappearance of the compatibility effect after these trials. This observation was not modulated by the cognitive load, *F* < *1*. Nevertheless, it increased through blocks, *F*_(1, 21)_ = 5.5, *p* < 0.05, $\eta_{p}^{2}$ = 0.21. But taken separately, the compatibility effect computed after an incompatible trial, *F*_(1, 21)_ = 2.9, *p* > 0.1, $\eta_{p}^{2}$ = 0.12, or a compatible trial, *F*_(1, 21)_ = 3, *p* > 0.1, $\eta_{p}^{2}$ = 0.12, did not increase across blocks. The interaction of all these variables was not significant, *F* < *1*.

As with accuracy, the RT analysis indicates a higher compatibility effect after a compatible trial than after an incompatible trial, (38 vs. 4 ms), *F*_(1, 21)_ = 48, *p* < 0.0001, $\eta_{p}^{2}$ = 0.7. Cognitive load had no effect, *F* < *1*. On the other hand, the compatibility effect was different with time on task, *F*_(1, 21)_ = 9.4, *p* < 0.01, $\eta_{p}^{2}$ = 0.31, and the interaction of these variables showed a trend, *F*_(1, 21)_ = 4, *p* = 0.06, $\eta_{p}^{2}$ = 0.16. Although the interaction was not significant, we chose to explore whether a group effect was present. Thus, we separated the analysis according to the compatibility of the preceding trial. After a compatible trial, the compatibility effect remained stable with time on task for both groups, *Fs* < *1*. On the other hand, after an incompatible trial, this effect increased with time on task for participants in the HCL group (block 1: −5 ms, block 3: 11 ms), *F*_(1,11)_ = 7.3, *p* < 0.05, $\eta_{p}^{2}$ = 0.4, but this was not the case for the other group (block 1: 4 ms, block 3: 6 ms), *F* < 1.

To sum up, our results demonstrate no effect of cognitive fatigue on anticipatory control. There is a trend on RT showing that for the HCL group the compatibility effect after an incompatible trial seems to increase. However, these results are based on exploratory analyses and should be taken with caution.

### Correlations Between Objective and Subjective Measures

We assessed whether the effects we observed were correlated with subjective measures. To this end, we computed the difference of the EMG measures (i.e., correction ratio, pre-motor time, motor time, and incorrect activations rate) obtained during the first and the third block of the Simon task and we evaluated the correlation between these differences and the evolution over time of subjective measures. More specifically, we separated the analyses according to the scores obtained during the TLDB task and those obtained during the Simon task. We proceeded in this manner because sometimes subjective experience precedes behavioral alterations. Therefore, we suggested that the perceived effort or increase in subjective fatigue following completion of the TLDB task could correlate with the behavioral effects of cognitive fatigue observed during the Simon task. We postulated that neither the evolution over time of sleepiness nor alertness should correlate with performance decrements. Given the large number of behavioral-subjective associations which was tested, we have considered the results of analyses below a threshold of *p* < 0.0016 to be significant.

**Figure 7:**
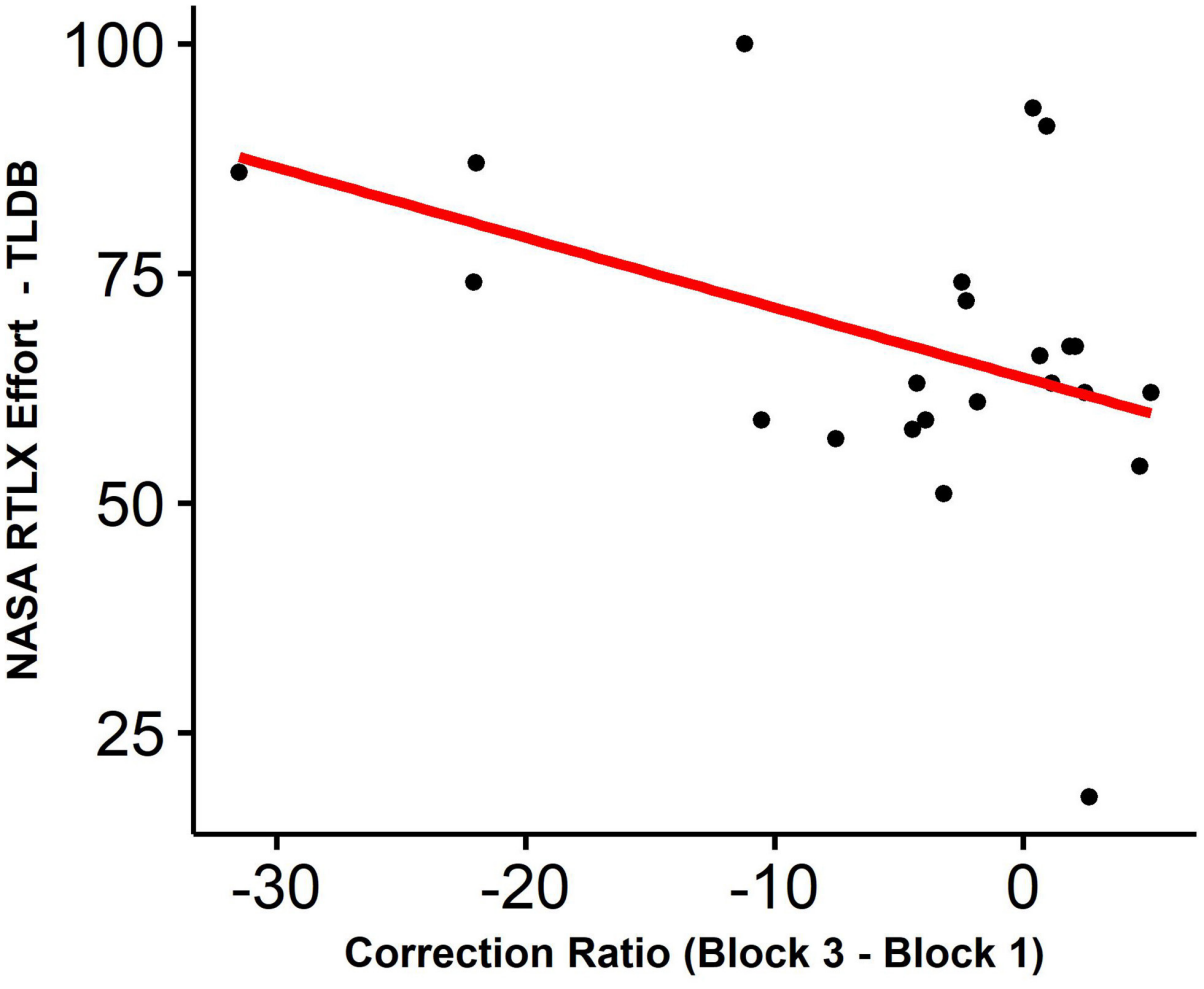
Correlation among (x) the difference between the correction ratio obtained in the third and the first block and (y) one subscale of the NASA RTLX that measures subjective effort related to the TLDB task.

We only observed a negative correlation between the reduction in the correction ratio and the subscale of the NASA RTLX measuring effort filled after the TLDB task (Pearson *r* = −0.42, *p* < 0.05). Thus, when participants reported a higher effort during the inducing task, they tended to be less effective to suppress the activation of the incorrect response during the Simon task (Figure 7). However, this result should therefore be considered with caution because the *p*-value was higher than the correction threshold we defined. Although the observed correlation was no longer present once the correction was applied, it confirms that separating perceived effort and subjective fatigue could be necessary. Importantly, neither the evolution over time of sleepiness or alertness correlated with performance decrements, as postulated.

## Discussion

The objective of this study was 2 fold. The first was to clarify the effect of cognitive fatigue on the two types of action control, online, and anticipatory control, during a conflict task. To achieve this, we relied on tools allowing a more detailed evaluation of these controls, the EMG and distribution analyses. This allows us to evaluate separately the automatic response activation and the response suppression, two mechanisms constituting online control. Moreover, the distinction between correct trials from those containing a partial error improves the accuracy of their evaluation. We observed during the two types of trials of the Simon task (i.e., compatible and incompatible) an increase in the number of incorrect activations as a function of time on task. This result was not attributed to an increase in the strength of the response capture. The presence of fast guesses rather suggests that the suppression mechanism was less engaged by participants and that they adopted a faster response strategy. Anticipatory control was not modulated by cognitive fatigue. The second objective was to evaluate the relation between objective and subjective measures, and we did not observe any. In the following sections, we will discuss these results and in particular the fact that they can be explained by the disengagement of effort.

### The Effect of Cognitive Fatigue on Action Control During the Simon Task

The results suggest that cognitive fatigue changed the way participants responded. Specifically, we observed that the suppression mechanism was less engaged with cognitive fatigue while the strength of the response activation remained the same.

To assess whether cognitive fatigue negatively impacted this suppression mechanism, we analyzed the correction ratio which reflects the efficiency of this mechanism. We observed for both groups that during incompatible trials the correction ratio decreased over time, suggesting that the suppression mechanism was less engaged with time on task. Importantly, this was not accompanied by an increase in the strength of automatic response activation. Indeed, the distribution of the number of incorrect activations obtained with the conditional incorrect accuracy function, especially on the first bins, remained stable over time. Since the reduction in the correction ratio over time was not accompanied by an increase in the strength of the automatic response activation, we can conclude that cognitive fatigue disrupts online control by impairing the suppression mechanism.

This result is consistent with those usually observed in the Go-noGo or stop-signal paradigms. Although these tasks rely on the involvement of different cerebral regions, in part because they do not require the choice of one response among several alternatives, these studies have frequently demonstrated that the suppression mechanism is less effective with cognitive fatigue, both at the behavioral and electrophysiological levels (e.g., Kato et al., 2009). However, as in conflict tasks, opposing results have been found (e.g., Falkenstein et al., 2002). It is possible that the strength of the automatic response activation was too large and exceeded the suppression capacity. For example, Freeman and Aron (2016) observed that when participants were fatigued, it was more difficult for them to inhibit a motor response when a high reward was associated with the stimulus, but this was not the case when the value of the reward was low (Freeman and Aron, 2016). It is assumed that assessing the strength of the response capture may provide clarifications in these paradigms. By differentiating these two mechanisms using EMG and distribution analysis, i.e., the strength of the automatic response activation and the response suppression mechanism, our results add new evidence in favor of an effect of cognitive fatigue on the suppression mechanism whereas no effect on the strength of the automatic response activation is observed.

Aside from the effects of cognitive fatigue on online control, we did not show that adaptation to the subsequent trial was less effective. The exploration of our results suggests a trend toward an increase in the Gratton effect over time but only for the group that performed the TLDB task with a high level of cognitive load. However, this result is not supported by a significant interaction, and should hence be considered as exploratory and be taken with caution. The low statistical power caused by our small sample size and the design of our experiment (i.e., between-subject comparison of the two groups) could be responsible for the absence of an observed effect.

### The Relation Between Objective and Subjective Measures

In this study, we evaluated the correlation between subjective fatigue, perceived effort, and EMG measures. In particular, we evaluated whether subjective measures assessed during a first task correlated with performance decrements observed during a second task. We observed that only perceived effort in completing the TLDB task correlated with the decrease in the correction ratio observed during the Simon task. However, this correlation should be considered with caution as it was no longer significant once the correction for multiple comparisons was considered. If it had been significant, this observation would have been consistent with motivational models of cognitive fatigue (Müller and Apps, 2019). These models suggest that cognitive fatigue increases the cost of effort and, if it becomes too high, participants stop making effort.

We observed no correlation between subjective fatigue and performance decrements. However, we did observe an increase in subjective fatigue, already during the TLDB task, while no decrease in performance was observed. This result is in line with Hockey's model, which indicates that subjective fatigue reflects the presence of a compensatory phenomenon which aims to maintain the level of performance (Hockey, 2013). This increase was independent of cognitive load. It continued to evolve in this direction during the Simon task and therefore increased throughout the study. The absence of difference according to the cognitive load can be explained by the fact that we have not been able to induce two different levels of cognitive load. Anyway, our results confirm the absence of a relationship between subjective and objective fatigue and show that EMG measures are not more sensitive than traditional behavioral measures.

Finally, we observed in this study an increase in sleepiness and a decrease in alertness. This result is not surprising given the duration of the task. However, it should be noted that these measures also did not correlate with performance decrements, which was consistent with our hypotheses.

### Cognitive Fatigue and Action Control: A Disengagement of Cognitive Effort

The previous results have shown that cognitive fatigue impaired only online control through a reduced involvement of the suppression mechanism. The implementation of this mechanism requires cognitive effort (Botvinick et al., 2001; Ridderinkhof et al., 2011; Ullsperger et al., 2014). Several results in our study suggest that with time on task, participants no longer engaged cognitive effort to the same extent. This assumption is consistent with models arguing that a decrease in the willingness to exert cognitive effort is associated with cognitive fatigue (Hockey, 2013; Massar et al., 2018; Müller and Apps, 2019).

We found that, for both groups, the number of incorrect activations increased with time on task regardless of trial compatibility. This increase observed in compatible trials means that participants may have changed their response strategy. More importantly, the presence of fast guess errors indicates that participants adapted their response strategy to respond more quickly. This suggests that they were no longer fully engaged in the task rather than an inability to perform the task, such as after a decrease of resources. In line with this, a speed-accuracy tradeoff has been observed. Indeed, in addition to the increase in the number of incorrect activations, a decrease in pre-motor time was observed with time on task. The evolution over time of this chronometric measure was observed regardless of the cognitive load level and trial compatibility. This association (i.e., reduction in the pre-motor time and increase in the number of incorrect activations) may suggest the presence of a speed-accuracy tradeoff. The presence of a speed-accuracy tradeoff has already been noted with cognitive fatigue. For example, Laurent et al. (2013) observed this speed-accuracy tradeoff during the last blocks of a switching task. With time on task, participants were faster but less accurate (Laurent et al., 2013). Importantly, this result is sometimes attributed to an effort disengagement, which is consistent with our observations. Indeed, this speed-accuracy tradeoff was not limited to incompatible trials, i.e., trials requiring effort. Since compatible trials were also concerned, a disengagement rather than an inability to exert effort may be suggested. This result has already been observed when disinvestment of effort was provoked, as in studies distributing a reward based on performance. In these studies, participants prefer to allocate effort on trials with high rewards but behaved inversely on trials with lower rewards. In this case, they exhibited avoidance behavior, choosing not to exert their effort and emphasizing speed over accuracy (Hübner and Schlösser, 2010; Otto and Daw, 2019).

Several studies have noted that cognitive fatigue leads to difficulties in sustaining cognitive effort. However, they have not always observed a speed-accuracy tradeoff (Wascher et al., 2014). In these studies, the RT was not separated into motor time and pre-motor time and errors into partials errors and “true” errors, which may explain some of the variability in results. Indeed, in our study, the two components showed an opposite trend (i.e. motor time increased and pre-motor time decreased with time on task). But when combined, a marginal increase with time on task was observed. Therefore, it is likely that this pattern of results was also present in previous studies, but that it was masked by the evaluation of conventional measures only.

However, the presence of a speed-accuracy tradeoff in our data could be questioned since it was not observed when we considered the motor time. Nevertheless, it is widely accepted that speed-accuracy tradeoff would mainly affect decision processes (Bogacz et al., 2010). Mathematical models of decision-making (e.g., Ratcliff and McKoon, 2008) argue that when speed is emphasized over accuracy, the amount of information accumulated to generate a response is faster due to a lower decision threshold, which could more easily lead to an incorrect decision and therefore to an error. The proposals of these models are supported by brain imaging studies (e.g., fMRI) showing, for example, that only a fluctuation in brain activity of the regions involved in decision-making (e.g., dorsolateral prefrontal cortex and pre-supplementary motor area) was observed when instructions emphasize speed (Forstmann et al., 2008; van Veen et al., 2008). Recently, observations have shown that the non-decision components, including motor components, could be also affected by this speed-accuracy tradeoff. For example, Spieser et al. (2017) observed that during a conflict task, when instructions emphasized speed, motor time was also reduced (Spieser et al., 2017). However, unlike our study, effort demand was not manipulated. Furthermore, the decrease in motor time may be masked by changes induced by the presence of cognitive fatigue. In contrast to pre-motor time, an increase in motor time with cognitive fatigue has been previously reported after sleep deprivation (Ramdani et al., 2013). A prolonged cognitive effort also generates an increase in sleepiness, which is also observed in our study. As proposed by Ramdani et al. (2013), sleepiness, especially induced by sleep deprivation, may decrease cortico-spinal excitation and muscle tension, which have been previously reported to affect motor time (Possamai et al., 2002; De Gennaro et al., 2007). Thus, sleepiness may have increased motor time in our study.

To conclude, all our results suggest that cognitive fatigue causes disengagement from cognitive effort. With cognitive fatigue, participants implemented online control to a lesser extent. Besides this result, they opted for an effortless response strategy by emphasizing speed over accuracy. These results are consistent with the motivational view of cognitive fatigue (Hockey, 2013; Müller and Apps, 2019). Although the observed correlation between the decrease in correction ratio and perceived effort during the TLDB task was no longer observed once the correction was applied, it is consistent with this interpretation.

## Limitations and Perspectives

Some limitations can be mentioned in this study. First, our sample size was small. The low statistical power could be responsible for the absence of difference between the two groups. However, it could also be explained by the proximity of the two TLDB tasks. We distinguished the two tasks by manipulating the cognitive load. Therefore, it is likely that the manipulation did not induce a large difference between the groups. The TLDB task, even in the simplest configuration, was still a complex dual-task. The small sample size also implies to be cautious with the interpretation of the results of the correlation analysis since it can be influenced by extreme values. In our opinion, our results fit well with motivational theories of cognitive fatigue. But we relied on indirect indicators. Assessing participant motivation to accomplish the task could have been important. Also, it might have been interesting to assess whether the response suppression and response capture mechanisms would have been modulated according to trial compatibility to broaden our understanding of the effects of cognitive fatigue on anticipatory control [see for e.g., Wylie et al. (2010) for such analyses]. However, this analysis was not possible because of the limited number of trials in our experiment. Our results showed that cognitive fatigue disturbs only online control rather than anticipated control. The design of our Simon task does not emphasize the use of anticipatory control. Thus, online control may have been primarily hampered because it was more widely used by participants. But this remains to be tested.

## Conclusion

To conclude, our results show the important contribution of EMG and distribution analyses. The measures they provide have led to a better understanding of the effect of cognitive fatigue on action control than traditional measures. This study demonstrated that cognitive fatigue leads to disengagement of effort resulting in impaired online and anticipatory control. Given the importance of adaptive capabilities for the safety of critical systems, these results are important as they provide a better understanding of the effects of fatigue on these capabilities.

## Data Availability Statement

The raw data supporting the conclusions of this article will be made available by the authors, without undue reservation.

## Ethics Statement

The studies involving human participants were reviewed and approved by Comité de Protection des Personnes Sud Méditerranée 1. The patients/participants provided their written informed consent to participate in this study.

## Author Contributions

All authors listed have made a substantial, direct and intellectual contribution to the work, and approved it for publication.

## Conflict of Interest

The authors declare that the research was conducted in the absence of any commercial or financial relationships that could be construed as a potential conflict of interest.

## Footnotes

^1^On the TLDB task, a composite score was computed using a weighted formula where the 2-back task and the judgment parity task represented, respectively, 65 and 35% of the total score. Borragán et al. (2017) made this choice to emphasize the information-retrieval component of the task.

^2^The task stopped six times (every 4 min) allowing participants to answer two questions. They had to evaluate the accuracy they obtained during the last block and to indicate their level of certainty about this evaluation. They reported their response on a visual analog scale. However, these results are not included in the present report.
